# Effectiveness of vaping prevention campaign messages on cognitive and behaviorally proximal outcomes among adolescents and young adults: a systematic review and meta-analysis

**DOI:** 10.3389/fpsyt.2026.1796793

**Published:** 2026-05-04

**Authors:** Aylin Akca Sumengen, Olayemi T. Adekeye, Gokce N. Cakir, Eyşan H. Savaş, Remziye Semerci Sahin, Mercy Mumba

**Affiliations:** 1 The University of Alabama, Capstone College of Nursing, Tuscaloosa, AL, United States; 2 Department of Nursing, TC Istanbul Gedik Universitesi, İstanbul, Türkiye; 3 Pediatric Oncology Department, Ospedale Infantile Regina Margherita, Turin, Italy; 4 School of Nursing, Koc Universitesi, İstanbul, Türkiye

**Keywords:** adolescents, campaigns, risk perception, susceptibility, vaping prevention

## Abstract

**Background:**

Vaping among adolescents has decreased but remains common among young adults, with ongoing concerns about nicotine health risks. Public health campaigns have increased vaping prevention messages, but evidence on their effectiveness, especially on cognitive and behavioral outcomes, is limited.

**Objective:**

This systematic review and meta-analysis aimed to synthesize recent experimental evidence on the effects of campaign-related vaping prevention messages on cognitive outcomes (e.g., knowledge, risk perception, perceived message effectiveness) and behaviorally proximal indicators (e.g., susceptibility to vaping and intentions to vape) among adolescents and young adults.

**Methods:**

A systematic search of five databases (Cochrane, PubMed, Scopus, CINAHL, and Web of Science) was conducted through January 2025. Thirteen Randomized Controlled Trials (RCTs) met the inclusion criteria, with six eligible for meta-analysis. The review adhered to PRISMA guidelines and was registered in PROSPERO ( CRD42025643032). The risk of bias was assessed using the RoB 2.0 tool. Meta-analyses were performed using a random-effects model to estimate pooled effect sizes.

**Results:**

The included studies (n=13) involved 11,235 participants aged 11–29 years. Most of the studies evaluated large-scale media campaigns or message-based interventions. Meta-analysis of six studies showed a statistically significant moderate effect on perceived vaping-related risk (Hedges’ g = 0.254; 95% CI: 0.187–0.321), with zero heterogeneity (I² = 0.0%). Due to the small number of studies, publication bias testing was exploratory. For behaviorally proximal outcomes, subgroup analyses revealed a statistically significant protective effect on vaping susceptibility (g = -0.166; 95% CI: -0.256 to -0.076; I² = 0.0%), while effects on intentions to vape were non-significant and heterogeneous (g = -0.089; I² = 62.3%). Narrative synthesis indicated consistent improvements in knowledge, attitudes, and perceived message effectiveness, particularly for messages emphasizing health harms and chemical exposure.

**Conclusion:**

Campaign-related vaping prevention messages demonstrate consistent and robust effects on cognitive outcomes and susceptibility to vape, but yield heterogeneous and context-dependent effects on intentions. These findings underscore the importance of strategic message design and thematic framing while highlighting the need for longitudinal and population-level studies to determine whether cognitive shifts translate into sustained behavioral change.

**Systematic review registration:**

https://www.crd.york.ac.uk/PROSPERO/view/CRD42025643032, identifier CRD42025643032.

## Introduction

Adolescent e-cigarette use has emerged as a major global public health concern (1). In the United States, although recent findings from the National Youth Tobacco Survey indicate a decline in youth vaping, more than 1.6 million middle and high school students remain current e-cigarette users (2). After a sharp rise in use during the mid-to-late 2010s (3), regulatory actions and public health interventions have contributed to recent declines (4, 5), yet adolescent vaping continues to pose a substantial public health challenge (6, 7). Any nicotine product use, especially vape and related products, among high school students, had peaked at 31.4% in 2019 (8). Subsequently, current vape use among high school students declined to 14.1% in 2022, 10.0% in 2023 (9) and 7.8% in 2024 (10). Despite this promising progress, vaping among young adults remains high at 15.5% (11). These trends indicate meaningful progress in reducing adolescent vaping while underscoring the continued public health relevance of prevention efforts, particularly for young adults (1, 8). Beyond prevalence trends, the health consequences of vaping among adolescents and young adults are substantial. Nicotine exposure during this period poses serious health risks, including adverse neurodevelopmental effects, increased addiction vulnerability, and disruption of neural circuits involved in impulse control and learning (2, 11–13). Vaping has also been linked to impaired respiratory function (14) and a higher likelihood of subsequent use of other tobacco products (15). As noted in Prochaska et al. (16), nicotine delivery from devices like JUUL can reach levels equivalent to multiple cigarettes per pod (approximately 14–33 mg per pod, comparable to 13–30 cigarettes), underscoring their high addictive potential.

Key factors driving vaping uptake among youth include appealing flavors, the pervasive influence of the media, and peer dynamics (17–19). These factors shape adolescent behavior through distinct pathways. Mass media and social networks primarily act as broad informational channels, frequently exposing youth to pro-vaping marketing. Concurrently, peer influence operates as a dynamic social mechanism; depending on the prevailing attitudes within a social group, interpersonal interactions can either normalize e-cigarette use or serve as a protective barrier through anti-vaping social norms (20, 21). Pro-vaping marketers often capitalize on this intersection by leveraging “peer voices” and social influencers across digital channels to target youth behavior (16). To counteract the saturation of pro-vaping content within these media environments, public health authorities increasingly utilize these same platforms to deliver targeted prevention strategies (22). Within this context, a preventive measure or message terms generally refers to strategically designed content, such as short narrative videos, graphic warning labels, or targeted social media posts, aimed at altering cognitive and behavioral trajectories regarding e-cigarettes (13, 23). Furthermore, evaluating the effectiveness of these measures relies on a clear conceptualization of exposure. In digital and mass media environments, exposure can range from passive scanning to active engagement (24). For an adolescent to be successfully reached, they typically must interact with the content long enough to process its core cue (e.g., viewing a 30-second video (7) or reading a graphic warning (25). This functional requirement highlights the predominantly short duration of most media-based interventions and directly informs how their cognitive and behavioral effectiveness is evaluated (13, 23). Prior studies underscore the importance of such communication strategies (26, 27). Organizations like the Truth Initiative have launched nationwide campaigns to discourage vaping among young individuals (22), while local efforts such as “Rethink Vape” have conveyed e-cigarette risks to youth (28). These initiatives suggest that prevention messages may more reliably influence beliefs and perceptions than immediate behavioral outcomes, underscoring the need to examine both domains simultaneously. Understanding the overall effectiveness of campaigns and related messages on both cognitive perceptions and risky behaviors is crucial for developing new, evidence-based anti-vaping communication strategies (7, 13, 29). These findings highlight the importance of assessing the effectiveness of current campaign-related vaping prevention messages.

Studies about vaping prevention messages suggest that messages emphasizing nicotine addiction, chemical harms, and graphic health consequences are perceived as more salient and effective by adolescents (19, 30). Qualitative findings indicate that messages emphasizing health risks can elicit strong emotional reactions and increase vaping-related risk perceptions (31). Several reviews have summarized experimental evaluations of these messages (13, 23, 26, 32), most notably Ma et al. (13), which offered an important and timely synthesis of message-level effects on various outcomes, such as risk perception, knowledge, and susceptibility. Since the publication of that review, the vaping prevention landscape has continued to evolve rapidly, with the emergence of new campaigns and additional empirical studies published in recent years. As the field expands, it has also become critical to clearly delineate the boundaries of behavior change interventions. While the broader spectrum of anti-vaping communication encompasses both primary prevention and cessation (23), these strategies target fundamentally different populations, such as susceptible non-users versus heavily nicotine-dependent users (23, 33) and operate through distinct psychological mechanisms (34). To maintain methodological homogeneity and prevent the obscuring of specific message effects, there is a need for a comprehensive synthesis that focuses exclusively on primary prevention (13). Furthermore, previous syntheses have frequently aggregated distinct behavioral indicators, potentially masking the nuanced psychological responses to prevention messages. Distinguishing between general susceptibility and definitive behavioral intentions is essential to fully understand how messages influence youth. Building on this foundational work, there is a need for a comprehensive synthesis that not only quantifies message-level effects but also integrates qualitative insights to inform the development of more effective, youth-focused vaping prevention campaigns. Accordingly, the purpose of the present systematic review and meta-analysis is to synthesize the most recent evidence from the last five years by combining a systematic review with quantitative meta-analysis, with a focus on experimentally evaluated campaign-related message exposure, in order to provide an updated assessment of how campaign-related vaping prevention messages influence both cognitive outcomes (e.g., knowledge, risk perception, perceived message effectiveness) and behaviorally proximal indicators (e.g., susceptibility and intentions) among adolescents and young adults.

## Method

### Study design

This systematic review and meta-analysis aimed to evaluate studies from the last five years assessing the effects of vaping prevention campaign-related message exposure on vaping intentions, risk beliefs, knowledge, and behavioral indicators among adolescents and young adults. The Preferred Reporting Items for Systematic Reviews and Meta-Analyses (PRISMA) guidelines were followed in conducting this systematic review and meta-analysis (35). The study protocol was also registered in the PROSPERO International Prospective Register of Systematic Reviews (CRD42025643032) (36). This study adheres to the rigorous seven-stage methodological framework established by Tricco et al. (37) and Langlois et al. (38) to ensure transparency and reliability in the research process. The stages included needs assessment and topic selection, study development, literature search, screening and study selection, data extraction, risk of bias assessment, and data synthesis and meta-analysis. The stages encompass a comprehensive approach, starting with a needs assessment and topic selection, which is particularly relevant given the alarming rise in vaping prevalence and its associated health risks (39, 40).

### Search strategy

A subject librarian performed a comprehensive literature search across Cochrane, PubMed, Scopus, CINAHL, and Web of Science databases from November 2024 to January 2025. Only studies published in English were included in the study, and there was no year limit for searching the databases. The keywords” #1 “Adolescent”[Mesh] OR adolescent OR Teenager OR Teen OR Youth OR Teen OR teenage* OR “Young Adult”[Mesh] OR “Young Adult”; #2 “Vaping”[Mesh] OR vaping OR Ecigarette OR ECig OR Vape OR “E-Cigarette” OR “E-Cig” OR “Electronic Cigarette”; #3 “Social Media”[Mesh] OR advert* OR “Mobile Social Media” OR “social media” OR “Mass Media”[Mesh] AND “mass media” OR “Broadcast Media” OR “Printed Media” OR “Ads” used in the review of databases included terms related to the target population (adolescents and young adults), vaping-related behaviors (vaping, e-cigarettes, electronic cigarettes), and media exposure (social media, advertisements, mass media, and printed media). To ensure comprehensive coverage, a supplemental search using the terms “Electronic Nicotine Delivery Systems” OR “ENDS” (combined with media/message-related terms) was executed on March 1, 2026, which identified two additional records. After full-text screening, neither study met the predefined inclusion criteria (one focused on program acceptability only, and the other evaluated harm-reduction messaging for smokers rather than prevention). Thus, no additional eligible studies were identified from this supplemental search. Boolean operators (AND, OR) were used to optimize the search strategy. The keywords used according to the databases are included in **Supplementary File S1**.

Inclusion and exclusion criteria: Inclusion and exclusion criteria were determined by patient/population, intervention, comparison, and outcomes (PICOS) (41).

- Population: Adolescents and young adults.
- Interventions: Used campaign-related messages to deliver vaping prevention information through mass media, social media, or other channels aimed at reducing susceptibility, changing behaviors, or increasing knowledge about vaping risks.
- Comparison: Control groups receiving no intervention, traditional tobacco prevention programs, or general health education programs that do not specifically target vaping.
- Outcomes: Susceptibility to vaping, intentions to vape, vaping attitudes, risk perceptions, perceived message effectiveness (PEM), reported vaping behaviors, and knowledge about vaping risks.
- Study Design: Randomized controlled trials (RCTs), including cluster-randomized designs, and pilot experimental studies (preliminary evaluations assessing feasibility or initial effectiveness) that assess the effectiveness of vaping prevention campaign–related message interventions and their outcomes.

The exclusion criteria for this study were studies that: (i) did not assess vaping prevention interventions; (ii) evaluated school-based programs focused on curriculum delivery rather than media messaging; (iii) lacked data on susceptibility, intentions, attitudes, perceived harm, vaping behavior, or knowledge; (iv) non-randomized or observational studies without experimental message exposure, including descriptive cross-sectional, cohort, retrospective analyses, case reports, summaries, and pharmacological treatment studies; (v) published more than five years ago; (vi) focused primarily on cessation instead of prevention; and (vii) lacked full-text access or were non-English studies.

### Study selection

**Figure 1:**
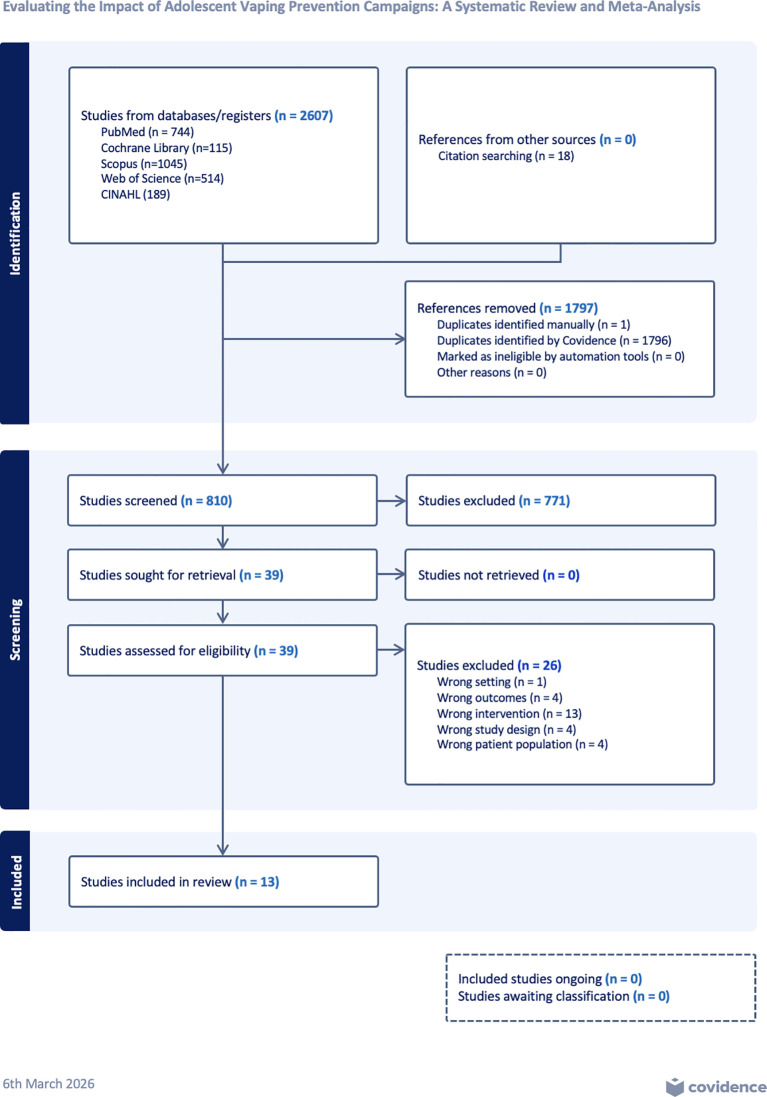
PRISMA flow diagram.

The study selection process followed a systematic and rigorous approach, as outlined in the PRISMA flow diagram (**Figure 1**). The COVIDENCE program (42) was utilized to facilitate data management and streamline the screening process. Two research team members independently screened all titles and abstracts to determine eligibility based on the inclusion criteria. Studies that met the initial eligibility criteria were then subjected to a full-text review conducted independently by two authors. If both reviewers agreed to exclude the study, it was removed from further consideration. In cases of disagreement, a third reviewer provided an independent assessment to reach a final consensus. As shown in **Figure 1**, the database searches (including the supplemental search) resulted in a total of 2,607 records prior to deduplication. Following the removal of 1,797 duplicate records, primarily identified using Covidence, 810 studies remained for title and abstract screening. At this stage, 771 of them were excluded based on the study’s eligibility criteria. A total of 39 full-text articles were sought for retrieval and assessed for eligibility. Of these, 26 studies were excluded for the following reasons: wrong intervention (n = 13), wrong outcomes (n = 4), wrong study design (n = 4), wrong patient population (n = 4), and wrong setting (n = 1). Ultimately, 13 studies met all inclusion criteria and were included in the systematic review, with six studies contributing data to the meta-analysis (**Figure 1**).

### Data extraction

After identifying the eligible studies, data were systematically extracted and organized to ensure consistency and accuracy. Key summary information from each selected study was compiled into a structured data extraction table. The extracted variables included the author(s), year of publication, country of study, sample size, participant age range, study design, types of interventions used in both the experimental and control groups, measurement methods, study outcomes, and conclusions, as shown in **Table 1**.

**Table 1:** Characteristics of the included studies.

Author/ Year/ Country	Population	Design	Type of Intervention	Interventions		Measurement	Results/Outcomes	Conclusion
				Experimental	Control			
Cartujano-Barrera et al., 2022, USA (25)	n=362 Black and Latino adolescents (ages 12–17).	RCT	Graphic message-based vaping prevention Participants were randomized into four groups, each receiving a specific graphic message	Graphic messages: Health Rewards: "Dying for a vape? It hurts more than you know" Financial Rewards: "Don't let your money vaporize away" Autonomy: "Vaping companies are targeting Black and Latino teens. Your life matters." Social Norms: "Just because vaping is common doesn’t mean it’s cool."	No control group (4 active arms)	-Susceptibility to Future Vaping: 3-item scale. -Message Satisfaction: 2-item scale. -These tools were adapted from previous studies.	-Susceptibility to Future Vaping: The health rewards message led to the greatest reduction in susceptibility (p = 0.125). -The social norms (p = 0.687) and autonomy (p = 0.435) messages also showed slight reductions. -The financial rewards message slightly increased susceptibility (p= 1.00). -Satisfaction with Messages: The health rewards message had the highest satisfaction (69.3% reported being satisfied or very satisfied) and recommendation rate (94.3% would share with a friend). -The financial rewards message had similar satisfaction (68.8%) and recommendation rates (90.3%). -The autonomy message had the lowest satisfaction (34.7%) and recommendation rates (70.6%).	The study found that graphic messages designed for Black and Latino adolescents had a modest but non-significant impact on reducing susceptibility to vaping. The health rewards message was the most effective, while the financial rewards message unexpectedly increased susceptibility.
England et al., 2021, USA (28)	n=415 adolescents aged 11–19 years (M age = 14.78). Southeastern Virginia. Final sample: 268 participants after data cleaning.	RCT	Community-driven risk communication campaign (The Rethink Vape campaign) targeting e-cigarette prevention using video ads, microsites, and social media.	Participants viewed final Rethink Vape visuals (e.g., campaign video ads, screenshots from the microsite) focusing on: What’s in the vapor? (chemicals in e-cigarettes). Health risks (e.g., lung disease, addiction). Big Tobacco (links to manipulative marketing strategies)	Participants viewed a different public health campaign, Rev Your Bev, which educated teens on the sugar content of drinks.	-Vaping Knowledge: 15-item knowledge scale. -Risk Perception (Risk Behavior Diagnosis Scale): 4-item Risk Behavior Diagnosis Scale. -Susceptibility to Threat: 3-item -Response Efficacy: 1-item -Intentions Not to Vape: 2-item Likert scale. -Experimentation Intent: 1-item -Expectancy: 3-item Likert scale -Addiction Belief: 1-item scale.	-Vaping Knowledge: Significant increase in knowledge after intervention (p < 0.001). -Risk Perception: Increased perceived harm of vaping (p < 0.001). -Susceptibility to Threat: Increased perceived personal risk (p < 0.001). -Response Efficacy: Improved belief that avoiding vaping reduces harm (p < 0.05). -Intentions Not to Vape: Strengthened resolve to avoid vaping (p < 0.001). -Experimentation Intent: Decreased likelihood of trying vaping, but not significantly (p > 0.05). -Addiction and Harm Expectancies: Increased beliefs about vaping’s addictiveness (p < 0.01) and health risks (p < 0.001). -Addiction Belief: Reduced belief that vaping is easy to quit (p < 0.05).	The Rethink Vape campaign effectively increased knowledge, risk perceptions, and anti-vape intentions among adolescents. Findings suggest that youth-focused, digital anti-vaping campaigns can positively shift beliefs and susceptibility to vaping. Further research should assess long-term behavior change and wider implementation across different populations.
Evans et al., 2024, USA (43)	n=1491 AYAs aged 18–24 years; stratified into based on use status. Final sample for follow-up: 854 participants.	RCT	Social media-based anti-vaping campaign (Truth Initiative) using 15-second video ads focusing on anti-industry beliefs about vaping	Participants were randomized into four treatment arms with increasing ad exposures: Arm 1: 4 video impressions. Arm 2: 8 video impressions. Arm 3: 16 video impressions. Arm 4: 32 video impressions.	No intervention	-Vaping Intentions: 2-item 5-point Likert scale, adapted from prior tobacco control research. -Anti-Industry Beliefs: 2-item 5-point Likert scale, measuring attitudes toward vape companies. -Self-Reported Advertisement Exposure: 4-item scale, averaging perceived exposure to intervention ads.	-Vaping Intentions: Social media intervention significantly reduced vaping intentions among current vapers (p = .04), but not in the full sample or other subgroups. -Anti-Industry Beliefs: Increased among current vapers (p = .04), but no significant effect in the full sample. -Self-Reported Advertisement Exposure: No significant effect of treatment on reported ad exposure (p > .05).	The findings highlight the potential of targeted digital campaigns for vaping prevention, but further research is needed on optimizing exposure levels and long-term effects.
Kalaji et al., 2022, USA (44)	n=1,016 YYAs aged 18–25 years	RCT	E-cigarette warnings targeting YYA and advertising health message strategies	Participants were randomly assigned to one of nine conditions based on warning type and ad message: Warning Types: FDA warning (Nicotine is an addictive chemical), YYA warning (This product is especially harmful to youth and young adults. It may cause mood disorders and damage to the brain.).	Participants exposed to a non-related control ad promoting smartphones, with similar layout but no reference to vaping.	-Affective Responses: 8-item positive and negative affect scale (5-point Likert scale). -E-cigarette Risk Perceptions: 6-item risk belief scale (4-point Likert scale). -Cognitive Elaboration: 2-item self-reported scale (4-point Likert scale). -Willingness to Vape: 3-item susceptibility scale (4-point Likert scale). -The tools were based on relevant literature.	-Affective Responses: The YYA warning increased negative affect (p < 0.001) and reduced positive affect (p < 0.001). -E-cigarette Risk Perceptions: The YYA warning increased youth-specific risk beliefs (p < 0.001), while the FDA warning had no effect (p > 0.10). -Cognitive Elaboration: The YYA warning increased cognitive elaboration (p < 0.001). -Willingness to Vape: No significant effect of warnings or advertising messages on willingness to vape (p > 0.05).	The study found that youth-focused warnings were more effective than the FDA-mandated warning in increasing negative affect, strengthening risk beliefs, and promoting cognitive elaboration about vaping harms. However, warnings did not significantly reduce willingness to vape.
Kieu et al., 2024, USA (45)	n=1,348 adolescents aged 13–17 years	RCT	Public health ads from FDA’s "The Real Cost" vaping prevention campaign focusing on health harms, addiction risks, and normative pressure.	Participants were exposed to three 30-second ads per week over three weeks (nine ads total). Health Harms Arm: Ads focused on vaping’s toxic chemicals and lung damage. Addiction Arm: Ads emphasized nicotine addiction and its consequences.	Neutral videos with no specific health messaging	-Susceptibility to Vaping: 3-item Likert scale (1-4). -Attitudes Toward Vaping: 3-item Likert scale (1-5). -Health Harm Risk Beliefs: 3-item Likert scale (1-5). -Addiction Risk Beliefs: 3-item Likert scale (1-5). -Injunctive Norms: 3-item Likert scale (1-5). -Negative Affect: 3-item Likert scale (1-5). -Cognitive Elaboration: 3-item Likert scale (1-5). -These tools were adapted from previous studies	-Susceptibility to Vaping: Exposure to The Real Cost ads reduced susceptibility (p < 0.01). -Attitudes Toward Vaping: Ads led to more negative attitudes (p < 0.01). -Health Harm Risk Beliefs: Increased beliefs in vaping-related health harms (p < 0.01). -Addiction Risk Beliefs: Strengthened beliefs in the addictive risks of vaping (p < 0.01). -Injunctive Norms: Increased perception that important others discourage vaping (p < 0.01). -Negative Affect: Ads evoked stronger negative emotions (p < 0.01). -Cognitive Elaboration: Increased thinking about the harms of vaping (p < 0.05).	The *Real Cost* ads effectively reduced susceptibility to vaping by increasing negative attitudes, strengthening health and addiction risk beliefs, and enhancing injunctive norms against vaping. The ads also evoked negative emotions and encouraged deeper thinking about vaping harms.
Kowitt et al., 2023, USA (46)	n=623 adolescents (ages 13–17), 53% female, 65% white, 19% Hispanic.	RCT	Public health video interventions focusing on smoking and vaping prevention	1. Smoking prevention video ad (FDA’s "The Real Cost" campaign): focused on health harms (3 ads) and addiction (3 ads). 2. Vaping prevention video ad (FDA’s "The Real Cost" campaign)	1. Neutral control video about smoking 2. Neutral control video about vaping	-Susceptibility to Vaping and Smoking: 3-item 4-point Likert scale -Negative Attitudes Toward Vaping and Smoking: 1-item 5-point Likert scale -Perceived Likelihood of Harm: 1-item 5-point Likert scale -Message Reactions (Attention, Negative Affect, Cognitive Elaboration, Avoidance, Reactance): Each measured using a 1-item 5-point Likert scale -These tools were adapted from previous studies	-Susceptibility to Vaping: No significant change (p=0.07). -Negative Attitudes Toward Vaping: More negative compared to control (p = 0.009). -Perceived Likelihood of Harm from Vaping: No significant change (p = 0.09). -Message Reactions: Increased attention (p < 0.001). Increased negative affect (p < 0.001). -Increased cognitive elaboration (p < 0.001). -No significant change in avoidance (p = 0.60). -No significant change in reactance (p = 0.54).	This experiment found that The Real Cost smoking prevention ads had beneficial spillover effects by increasing negative attitudes toward vaping and raising perceived vaping harm.
Lazard et al., 2021, USA (47)	n=928 adolescents (ages 15-18)	RCT	Social media-based vaping prevention messages	Visual-based messages: Images illustrating e-cigarette risks (e.g., lung damage, chemicals, mood effects) Quiz format: True/False format with immediate feedback Text-only messages	No intervention	-Message Reactions 3-item University of North Carolina PME Scale (5-point Likert scale). -Cognitive Elaboration: 1-item self-reported scale (5-point Likert scale). -Affect: 1-item 7-point Likert scale -E-cigarette Knowledge: 8-item true/false -Beliefs About E-cigarette Risks: 7-item Likert scale -Message Sharing Intentions: Self-reported sharing scale.	-Message reactions: Increased across all message formats (p < 0.001). -Cognitive Elaboration: Messages led to significant increases in thinking about vaping risks (p < 0.001). -Affect: Negative affect was highest for harmful chemicals and lung damage messages (p < 0.001). -E-cigarette Knowledge: Social media messages improved knowledge (p < 0.001). Beliefs About E-cigarette Risks: Strengthened beliefs about vaping harms (p < 0.001). -Message Sharing Intentions: 79% of adolescents reported willingness to share messages, positively associated with PME (p < 0.001).	Social media messages effectively increased knowledge and beliefs about e-cigarette harms. The UNC PME Scale confirmed high perceived effectiveness, and beliefs about vaping risks were significantly strengthened. Messages focusing on lung damage and harmful chemicals had the strongest impact.
Llanes et al., 2023, USA (14)	n= 1,229 AYAs aged 18–25 years (M = 21.4 years; SD = 2.2)	RCT	Social media-based campaign experimental intervention using Instagram posts about e-cigarette or vaping-associated lung injury (EVALI).	Participants viewed Instagram posts with varying image valence (positive or negative), tailored to their assigned condition.	No control group	-Self-reported ratings -Perceived Harmfulness of Vaporizer Products: 4-item scale (1-4) -Perceived Risk of Vaping (4-item Likert scale, 1-4) -Intentions to Use Vaporizers (1-item Likert scale, 1-4) -Susceptibility to Vaping: Assessed among never-users.	-Perceived Harmfulness of Vaporizer Products: Increased perceived harm for nicotine vaporizers (p < 0.05) and cannabis vaporizers (p < 0.05). -Perceived Risk of Vaping: Negative images increased perceived risk of nicotine vaporizer use (p < 0.01), but no significant effect on cannabis vaporizer risk perceptions (p > 0.05). -Intentions to Use Vaporizers: Negative images decreased intentions to use nicotine vaporizers (p = 0.02), but no significant reduction in cannabis vaporizer use intentions (p > 0.05). -Susceptibility to Vaping: Negative imagery in social media posts about EVALI was linked to lower susceptibility to vaping among young adult never-users.	Negative imagery in Instagram posts about EVALI increased perceived harm, reduced intentions to use nicotine vaporizers, and effectively lowered susceptibility to vaping among young adult never-users but had no significant effect on cannabis vaporizers.
Noar et al., 2020, USA (48)	n=543 adolescents aged 13–17 years	RCT	Persuasive anti-vaping ads from the FDA’s "The Real Cost" campaign	Participants were exposed to two persuasive e-cigarette prevention video ads from the FDA’s "The Real Cost" campaign: Focused on addiction, harmful chemicals, and vaping’s health risks.	Control group viewed two informational videos from Mayo Clinic: Covered e-cigarette risks using “news style” visuals.	-PME: Message Perceptions: 6-item 5-point Likert scale, -Effects Perceptions: 13-item 5-point Likert scale -Risk Beliefs About Vaping: 9-item 5-point Likert scale -Attitudes Toward Vaping: 3-item 5-point Likert scale -Intentions to Vape: 3-item 5-point Likert scale -These tools were adapted from prior research	-Message Perceptions: The Real Cost ads scored higher than control (p < 0.001). -Effects Perceptions: The Real Cost ads scored higher than control (p < 0.001). -Risk Beliefs About Vaping: Higher among those exposed to The Real Cost ads (p < 0.001). -Attitudes Toward Vaping: More negative attitudes among those exposed to The Real Cost ads (p < 0.001). -Intentions to Vape: Lower intentions among those exposed to The Real Cost ads (p = 0.024).	Exposure to The Real Cost vaping prevention ads led to higher perceived vaping risks, more negative vaping attitudes, and lower vaping intentions compared to control messages.
Noar et al., 2022, USA (7)	n=1,514 adolescents aged 13–17 years	RCT	FDA’s "The Real Cost" e-cigarette prevention ads targeting health harms and nicotine addiction.	Health Harms Group: Ads focused on toxic substances and lung damage. Addiction Group: Ads emphasized nicotine addiction and its long-term consequences. Participants viewed three 30-second ads weekly for 3 weeks.	Participants viewed neutral videos about vaping (definitions, farming practices, etc.), consisting of black text on a white screen with a narrated voice.	-Susceptibility to Vaping: 3-item 4-point Likert scale -Susceptibility to Smoking: 3-item 4-point Likert scale -Message Reactions: Attention: 1-item 5-point Likert scale Negative Affect: 3-item 5-point Likert scale -Vaping-Related Outcomes: Cognitive Elaboration: 3-item 5-point Likert scale -Vaping Health Harm Risk Beliefs: 3-item 5-point Likert scale -Vaping Addiction Risk Beliefs: 3-item 5-point Likert scale -Vaping Attitudes: 3-item 5-point Likert scale Vaping Behavior: Self-reported past 7-day e-cigarette use -These tools were adapted from prior research	-Susceptibility to Vaping: Lower in The Real Cost groups compared to control (p < 0.001). -Message Reactions: Higher attention to ads (p < 0.001). -Higher negative affect (stronger emotional response) (p < 0.001). -Vaping Outcomes: Higher cognitive elaboration (p = 0.002). -Higher addiction risk beliefs (p = 0.006). -Higher health harm risk beliefs (p < 0.001). -More negative vaping attitudes (p < 0.001). -Lower vaping behavior (fewer days vaped per week) (p = 0.03).	This randomized clinical trial found that exposure to The Real Cost vaping prevention ads significantly reduced susceptibility to vaping and smoking, increased perceptions of vaping and smoking risks, and decreased self-reported vaping and smoking behavior.
Rohde et al., 2021, USA (49)	n=557 AYAs (aged 18–29 years	RCT	FDA’s "The Real Cost" e-cigarette prevention ads targeting addiction and health risks.	Participants viewed two 30-second FDA ads focused on nicotine addiction and vaping health risks. Ads were systematically developed for the campaign and designed to dissuade young people from vaping.	Participants viewed two Mayo Clinic informational ads (30 seconds each) with a "news style" format about e-cigarette risks.	- -PME: 6-item Likert scale (1-5) -Effects Perceptions: 10-item Likert scale (1-5) -Risk Beliefs About Vaping: 9-item Likert scale (1-5) -Intentions to Vape: 3-item Likert scale (1-5) -These tools were adapted from prior research	-PME: FDA The Real Cost ads scored significantly higher than control ads p < .001). -Effects Perceptions: FDA ads rated significantly higher than control (p < .001). -Risk Beliefs About Vaping: Higher in the FDA ad condition (p = .022). Intentions to Vape: -No significant difference between FDA and control groups (p = .858).	The findings suggest that PME and effects perceptions play distinct roles in shaping risk beliefs, with effects perceptions being a stronger predictor of risk beliefs and vaping intentions.
Rohde et al., 2022, USA (50)	n=623 adolescents aged 13–17 years	RCT	Text-based vaping warning campaign (based on FDA’s "The Real Cost" campaign) targeting specific health themes.	Adolescents were randomized into four warning message groups and viewed 3 messages from their assigned group. Nicotine Addiction Chemical Harms Lung Harms COVID-19 Harms	Control group viewed three messages about vape litter (e.g., “Vapes require cleanup, do not litter”).	-PME: 3-item 5-point Likert scale -Negative Affect (Fear): 1-item 5-point Likert scale -Attention: 1-item 5-point Likert scale -Anticipated Social Interactions: 1-item 5-point Likert scale -Message Novelty: 1-item 5-point Likert scale These tools were adapted from prior research	- PME: The chemical, lung, and COVID-19 harms warning themes were rated significantly higher on PME than both nicotine addiction and control (p < 0.05). Nicotine addiction was rated higher than control (p < 0.05). -Negative Affect (Fear): The chemical, lung, and COVID-19 themes elicited greater negative affect than nicotine addiction and control (p < 0.05). -Attention: The COVID-19, chemical, lung, and nicotine addiction themes were all rated as more attention-grabbing than control (p < 0.05). COVID-19 was also rated higher than nicotine addiction. -Message Novelty & Social Interactions: The COVID-19 harms theme was rated higher than both nicotine addiction and control for message novelty and anticipated social interactions (p < 0.05).	Adolescents perceived warning message themes about lung, chemical, and COVID-19 health effects of vaping as more effective than nicotine addiction. To discourage vaping, health messaging should expand beyond nicotine addiction to communicate a broader set of health effects
Sutfin et al., 2024, USA (51)	n = 586 (Phase II) AYAs aged 16–25 years	CRCT	Point-of-sale e-cigarette prevention ads focusing on nicotine addiction and health risks were displayed at gas stations and convenience stores.	Campaign Messages: Featured nicotine’s harmful effects on brain development and general e-cigarette risks. Displayed on gas pump toppers, store doors, cooler clings, and other prominent store locations.	Stores with no messages displayed served as the control condition.	-Chemical Knowledge: 2 items (assessing nicotine presence and harmless water vapor beliefs). -Outcome Expectancies: 7 items total on 5-point Likert scales (assessing health worry, health consequences, and nicotine inhalation). Relative Harm -Perception: 1-item 5-point Likert scale. -Attitudes Toward E-Cigarettes: 5-item 5-point semantic differential scale. -Worry About Nicotine Inhalation: 1-item 4-point Likert scale. -Behavioral Intentions to Vape: 3-item 4-point Likert scale. These tools were adapted from prior research.	-Message Exposure: Real-world message exposure was low (31.8%). -Intervention Effects: There were no significant differences in changes from baseline to follow-up between the intervention and control arms for chemical knowledge, outcome expectancies, attitudes, worry, or behavioral intentions (p > 0.05).	Point-of-sale messaging shows promise as a method for reaching adolescents and young adults, but low exposure rates in a crowded marketing environment limit its effectiveness. Longer campaign durations and additional channels may improve outcomes.

RCT, Randomized Controlled Trial; CDC, Centers for Disease Control and Prevention; NYTS, National Youth Tobacco Survey; CRCT, Cluster Randomized Controlled Trial; VR, Virtual Reality; SHA, Second Hand Aerosol; PME, Perceived Message Effectiveness; AME, Actual Message Effectiveness; CTV, Clearing the Vapor; vAA, Authors and Audiences; vMM, Messages and Meanings; vRR, Representation and Reality; ATI-V, Above the Influence (ATI), Above the Influence of Vaping; YRBS, Youth Risk Behavior Survey; FDA, Food and Drug Administration; YYA, Youth and Young Adults.

### Quality appraisal

**Figure 2:**
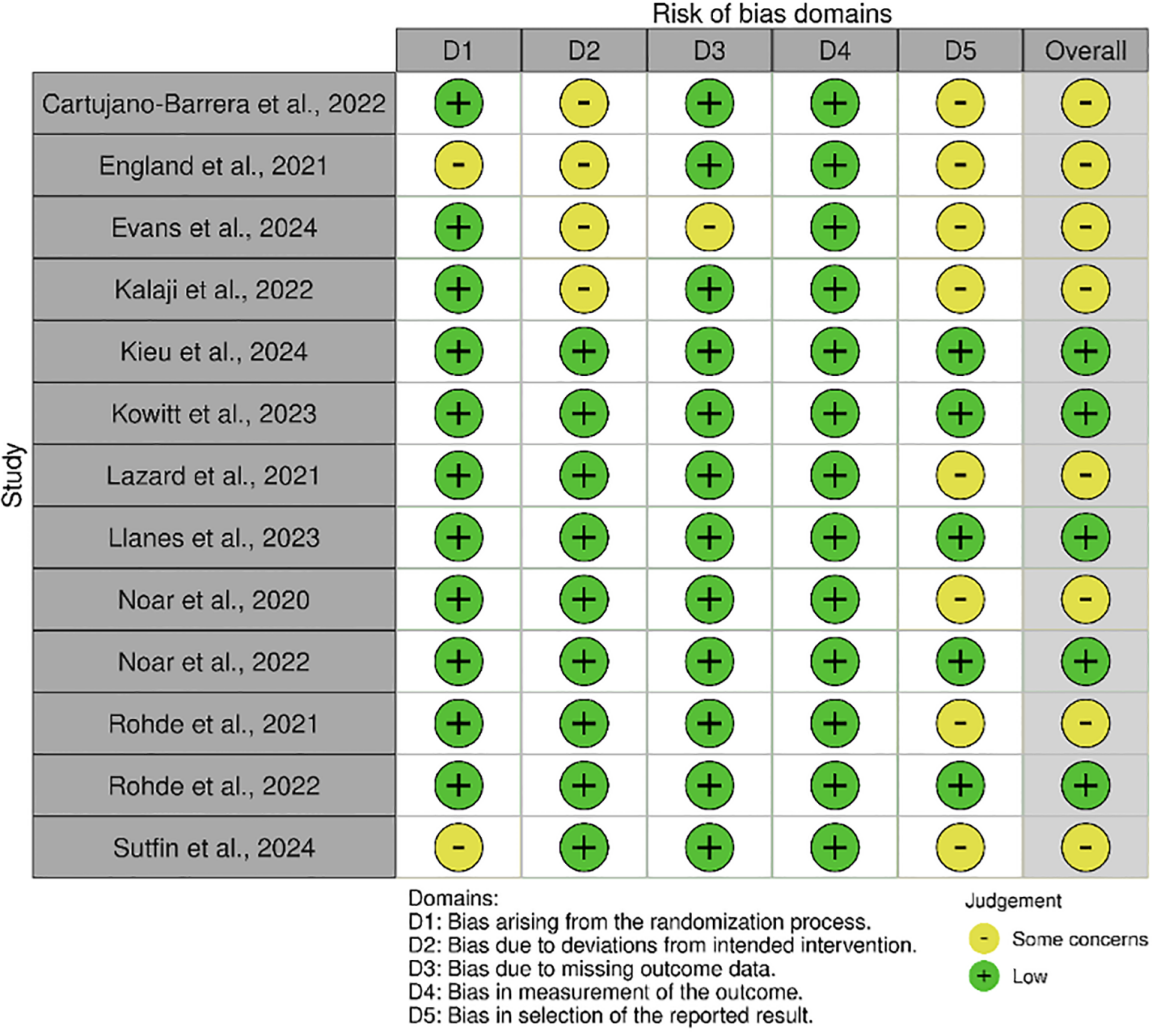
Quality assessment of the studies (RoB2).

Two researchers independently evaluated the included studies using established risk of bias assessment tools. Any discrepancies between the researchers were resolved through re-evaluation to reach a consensus. The RoB 2.0 tool was applied to assess five key domains: the randomization process, deviations from the intended intervention, missing outcome data, measurement of outcomes, selection of reported results, and overall risk of bias (52). Overall, the majority of included studies were judged to have a low risk of bias or some concerns, with the most common sources of potential bias related to the randomization process and deviations from intended interventions, while outcome measurement and reporting were generally assessed as low risk (**Figure 2**).

### Data synthesis and analysis

The meta-analysis was conducted using the Comprehensive Meta-Analysis software (53). The included studies’ mean, standard deviation, sample size, post-test, and p-values were utilized. The effect size (Hedges’ g and 95% confidence intervals [CI]) was calculated to determine the difference between the intervention group’s mean and the control group’s mean, divided by the combined standard deviation. Hedges’ g, similar to Cohen’s d, was interpreted using standard thresholds: 0.2 (small effect), 0.5 (medium effect), and 0.8 (large effect). Heterogeneity among studies was assessed using Cochran’s Q test (p <.10) and Higgins’ I² test, categorized as low (0-40%), moderate (30-60%), substantial (50-90%), and considerable (75-100%). An I² value greater than 50% was considered large enough to indicate significant heterogeneity. Additionally, statistical tests such as Orwin’s fail-safe N, Egger’s regression test, Tau coefficient, and Begg’s adjusted rank correlation test were applied to evaluate potential publication bias. Additionally, possible sources of heterogeneity or inconsistencies in effect size and direction across studies were examined (54, 55). Given the limited number of studies included in the meta-analyses (k < 10), formal statistical tests for publication bias were considered exploratory, as they may lack sufficient power to definitively rule out bias.

### Ethical considerations

As this study is a systematic review and meta-analysis, it did not involve direct human subject research and was therefore exempt from institutional ethical approval. However, to ensure research integrity, we verified that all primary studies included in our synthesis reported obtaining appropriate ethical approval from their respective Institutional Review Boards (IRBs).

## Results

### Study characteristics

The included studies collectively involved 11,235 adolescents and young adults and were conducted in the United States between 2020 and 2024. The age of participants ranged from 11 to 29 years, with eight studies focusing on adolescents (7, 25, 28, 45–48, 50), while five studies focused exclusively on young adults or mixed populations of adolescents and young adults (14, 43, 44, 49, 51). All included studies focused on vaping prevention campaign related message interventions. The majority of the studies (n=12) employed a RCT design (7, 14, 25, 28, 43–50), and one study (n=1) employed a cluster randomized controlled trial (CRCT) (51).

### Interventions for vape prevention

The 13 included studies explored a variety of interventions aimed at preventing vaping among adolescents and young adults. These interventions were broadly grouped into two categories: public health campaign related message interventions and graphic/message-based strategies.

Public Health Vape Prevention Campaign-Related Message Interventions: Six studies fell under this category (7, 28, 43, 45, 46, 48). The intervention usually involved short videos or digital advertisement strategies. Campaign themes included nicotine addiction, chemical exposure, lung damage, and vaping industry marketing deception. Many incorporated rigorous formative message testing, randomized exposure, and pre-post evaluations of attitudes, beliefs, and behavioral intentions. Five of the included studies examined variations of *The Real Cost* campaign messages (7, 45, 46, 48, 49). Additionally, England et al. (28) evaluated materials from the Rethink Vape campaign and described prominent message themes (e.g., ingredients/chemicals, health risks, and industry-related content), as well as adolescents’ reactions to these themes.

Graphic and Message-Based Interventions: Seven studies examined interventions that focused on the design, framing, and delivery format of anti-vaping messages, to assess campaign-related message effectiveness (14, 25, 44, 47, 49–51). These studies shared an emphasis on testing the persuasive potential of different thematic elements (e.g., chemical harm, lung damage, addiction), message tones (e.g., explicit vs. implicit), and delivery channels (e.g., visual warnings, point-of-sale displays, text messaging). Across these interventions, studies commonly examined differences in thematic framing and presentation features (e.g., health harms, chemicals/ingredients, addiction, imagery, and message tone) and assessed outcomes such as PEM and related cognitive responses. Three studies specifically tested message themes related to chemical exposure and health harm, finding these to be more effective than addiction-focused warnings (44, 49, 50). For instance, Rohde et al. (50) identified that messages about chemical and lung harms were rated as more persuasive than those centered on nicotine addiction, while Kalaji et al. (44) showed that youth-oriented warnings enhanced emotional responses and perceived relevance. Rohde et al. (49) further demonstrated that effect perceptions, how much a message is expected to change behavior, were more predictive of outcomes than general message impressions. In a real-world setting, Sutfin et al. (51) implemented a point-of-sale campaign at gas stations targeting adolescents with messages about nicotine’s effects on brain development. Cartujano-Barrera et al. (25) evaluated the immediate impact of culturally tailored graphic messages, though a single exposure did not produce statistically significant changes in adolescent susceptibility to vaping. All studies in this category highlighted the role of strategic content framing and tailored messaging in shaping adolescents’ responses to anti-vaping communication.

### Outcome measures

The majority of studies employed measurement tools that had been utilized in prior research. *Knowledge* was measured by England et al. (28), using a 15-item questionnaire developed specifically to assess vaping-related factual information shared in their campaign material (56). *Attitudes* toward vaping were assessed by Noar et al. (48) and Noar et al. (7) using 3- to 5-item Likert scales (4- or 5-point), adapted from Zhao et al. (57). However, Sutfin et al. (51) used a five-item semantic differential scale (e.g., bad-good, harmful-harmless) to assess attitudes toward e-cigarettes. *Risk beliefs* about vaping were typically assessed using 3- to 9-item Likert scales (4- or 5-point), with many studies adapting items from Brennan et al. (58)Sangalang et al. (59), and Wackowski and Delnevo (7, 48, 49, 51, 60). *Susceptibility to vaping* was commonly measured using a 3-item 4-point Likert scale (45, 46), adapted from the Expanded Susceptibility Index (61). *Behavioral intentions to vape* were typically assessed with 2- to 3-item Likert scales (4- or 5-point), adapted from prior tobacco prevention research (43, 51). Five studies measured *perceived message effectiveness (PME)* (47–51) using 3- to 6-item 5-point Likert scales, commonly used in pre-testing of FDA campaigns (7, 48, 62, 63).

### Narrative synthesis of outcomes

Knowledge and attitudes toward vaping: Two studies (28, 47) reported significant increases in knowledge, and four studies reported changes in anti-vaping attitudes (43, 45, 46, 48) among current users following exposure to vaping-related messages. While Evans et al. (43) found significant effects on attitudes and intentions specifically among current users, the other studies demonstrated these positive shifts across their broader adolescent samples. These studies demonstrated that educational campaigns effectively enhanced participants’ understanding of vaping-related harms while simultaneously shaping more negative attitudes toward vaping. Exposure to public health campaign approach led to statistically significant improvements in participants’ knowledge regarding the health risks associated with vaping. For example, studies assessing public health campaign messages found that participants exposed to prevention message had significantly higher scores about vaping-related harms compared to control groups (48). Similarly, experimental studies exposing youth to campaign video advertisements (43, 46), resulted in increased awareness of vape marketing tactics and negative shifts in vaping attitudes. However, the findings from Sutfin et al. (51) did not reach statistical significance for either knowledge or attitudes toward vaping, suggesting that real-world message exposure might produce more modest or context-dependent effects.

Risk perception and perceived consequences of vaping: The majority of the studies (n=7) (7, 14, 44, 45, 47–49) demonstrated that youth-targeted warning messages, public health campaigns, media literacy programs, and social media messages effectively heightened awareness of vaping’s health risks. In several of these studies, this increased risk perception co-occurred with more negative attitudes toward vape use and a lower likelihood of future vaping behavior. Risk perception refers to how individuals evaluate the potential dangers of vaping, including physical, mental, and social consequences. For instance, a study assessing youth-specific warning messages (44) found that participants who viewed warnings about brain damage and mood disorders from vaping were significantly more likely to believe in the severe health risks of e-cigarette use. Additionally, studies evaluating social media-based messages demonstrated that youth-targeted content highlighting the deceptive practices of vape companies (47) or organic posts about vaping-associated lung injury (EVALI) (14) strengthened participants’ beliefs about vaping risks, and in the case of Llanes et al. (14), reduced their intentions to vape in the future.

Perceived Message Effectiveness (PME): Several studies (47–51) examined PME to measure how persuasive participants found vaping prevention messages in discouraging use. The findings indicated that messages highlighting health risks and addiction were perceived as more effective compared to other themes. While PME captures participants’ perceptions of message persuasiveness, some studies also explored related constructs by differentiating between message perceptions and effect perceptions (48, 49). Message perceptions, evaluated in these studies, focused on cognitive processing and engagement (e.g., whether the ad grabs attention), whereas effect perceptions directly assessed participants’ judgments about a message’s potential to change their attitudes or behavioral intentions. Message reactions and PME, assessed in Lazard (47), evaluated both immediate cognitive/emotional responses and the messages’ perceived effectiveness in discouraging vaping.

Behavioral intentions and susceptibility to vaping: Behaviorally proximal outcomes, such as susceptibility and intentions to vape, are critical predictors of future product initiation and use (7, 45). However, unlike cognitive outcomes (e.g., risk beliefs or knowledge), susceptibility and intention outcomes tend to be more heterogeneous based on intensity of the exposure, target population, and specific study context (25, 49). Interventions with longitudinal exposure, have been associated with reductions in openness to vaping in some individual studies. For instance, Noar et al. (7)’s study resulted in significantly lower susceptibility to vaping among the target population. Similarly, England et al. (28) reported positive changes in behavioral indicator measures following exposure to Rethink Vape campaign related message materials. However, repeated delivery of social media messages showed nuanced effects on vaping intentions. A 60-day social media intervention trial with repeated exposures by Evans et al. (43) found a significant effect on lower vaping intentions only among current vapers and found no similar effect in the overall sample or among never users. Kalaji et al. (44) found that their study intervention did not influence future intentions to vape. Similarly, same type of experiments targeting university students found no significant difference in vaping intentions between the intervention and control groups (49). In studies focusing on vulnerable subgroups, single exposures failed to achieve statistical significance again (25). Despite minimal effects from a single exposure, certain message features slightly improved proximal outcomes. For instance, negative imagery in social media posts about EVALI was linked to lower susceptibility to vaping among young adult never-users. (14).

### Meta-analysis results

This meta-analysis, based on seven studies, examined whether exposure to campaign-related vaping prevention messages is associated with changes in adolescents’ perceptions of vaping-related risks and behaviorally proximal outcomes.

#### Risk perception

This meta-analysis included 3,641 participants across six studies. To assess whether the effect sizes varied across studies, heterogeneity was examined. The Cochrane Q statistic was 3.012 (p = 0.698), and the I² value was 0.00%, indicating zero heterogeneity among the included studies. Based on this result, a random-effects model was applied. Using the random-effects model, the average effect size was found to be 0.254 (medium effect size) (64), corresponding to a moderate standardized mean difference, with a 95% confidence interval ranging from 0.187 to 0.321, indicating a consistently positive effect across studies. Because the confidence interval does not include the null, the estimate suggests a statistically significant and reasonably precise increase in perceived vaping-related risk following message exposure. Furthermore, because the between-study variance (tau-squared) was estimated as zero, a prediction interval is not reported. This indicates that all studies in the analysis are assumed to share a common effect size with no dispersion of true effects, emphasizing the highly consistent nature of this finding. The positive effect size indicates that adolescents in the intervention group perceived a higher risk compared to those in the control group. Several methods were used to assess the risk of publication bias. Orwin’s fail-safe N was 1, meaning that 1 additional study with no effect would be needed to reduce the overall effect size to under 0.253. Rosenthal’s classic fail-safe N was 76, exceeding the commonly accepted threshold of 5k + 10 (where k is the number of included studies) (65), which in this case equals 40. While Begg and Mazumdar’s rank correlation test (tau = 0.200, p = 0.573) and Egger’s regression test (intercept = 0.684, p = 0.605) were both statistically non-significant, these formal tests were exploratory due to the small sample size (k = 6). Therefore, the absence of publication bias cannot be definitively confirmed. Additionally, Duval and Tweedie’s Trim and Fill method identified 1 missing study. The estimated effect size was slightly reduced after adjustment (g = 0.236), which further supports the stability of the result (**Table 2**).

**Table 2:** Meta-analysis results on risk perception.

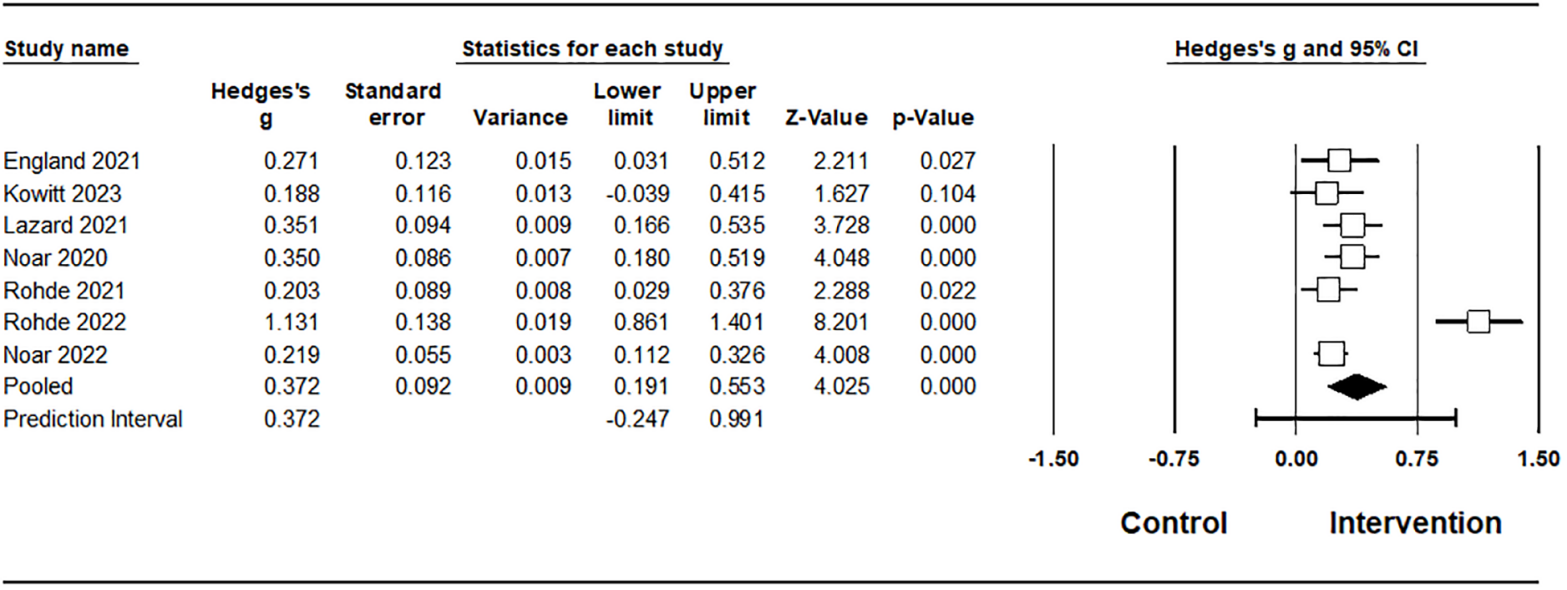

#### Behaviorally proximal outcomes

The meta-analysis synthesized data from five studies examining the effects of campaign-related vaping prevention message exposure on behaviorally proximal outcomes among adolescents and young adults, including susceptibility to vaping and intentions to vape. Across studies, a total of 3,182 participants were included in the pooled meta-analytic sample. Given conceptual and measurement differences between susceptibility and intention outcomes, these outcomes were not pooled into a single overall effect size. For intentions to vape, two studies were included. Under a random-effects model, the pooled effect size was small (Hedges’ g = -0.089), with a 95% confidence interval ranging from -0.286 to 0.107, indicating that the estimate was imprecise and not statistically significant. Heterogeneity for intention outcomes was substantial (I² = 62.3%), indicating considerable variability across studies. For susceptibility to vaping from three studies, the analysis showed that the random-effects pooled effect size was -0.166, with a 95% confidence interval ranging from -0.256 to -0.076, indicating a statistically significant protective effect. This finding is highly consistent across studies, as reflected by zero observed heterogeneity (I² = 0.0%). Due to the limited number of studies for each outcome, publication bias cannot be ruled out (**Table 3**).

**Table 3:** Meta-analysis results on behaviorally proximal outcomes..

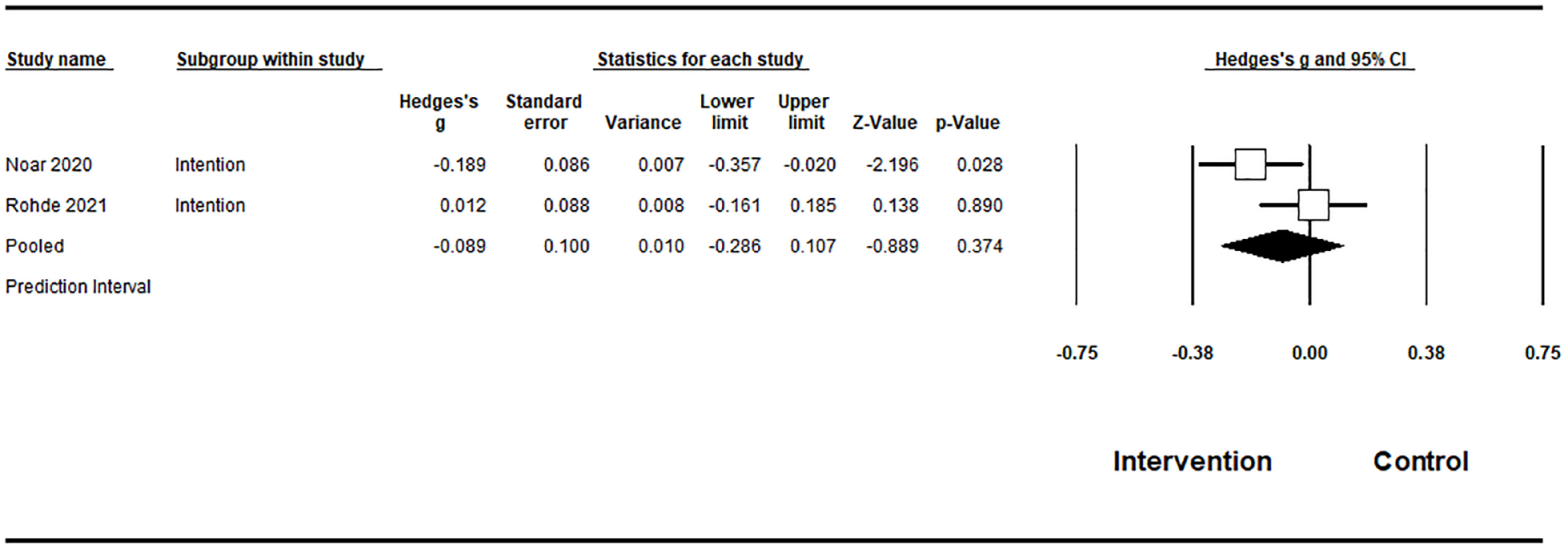
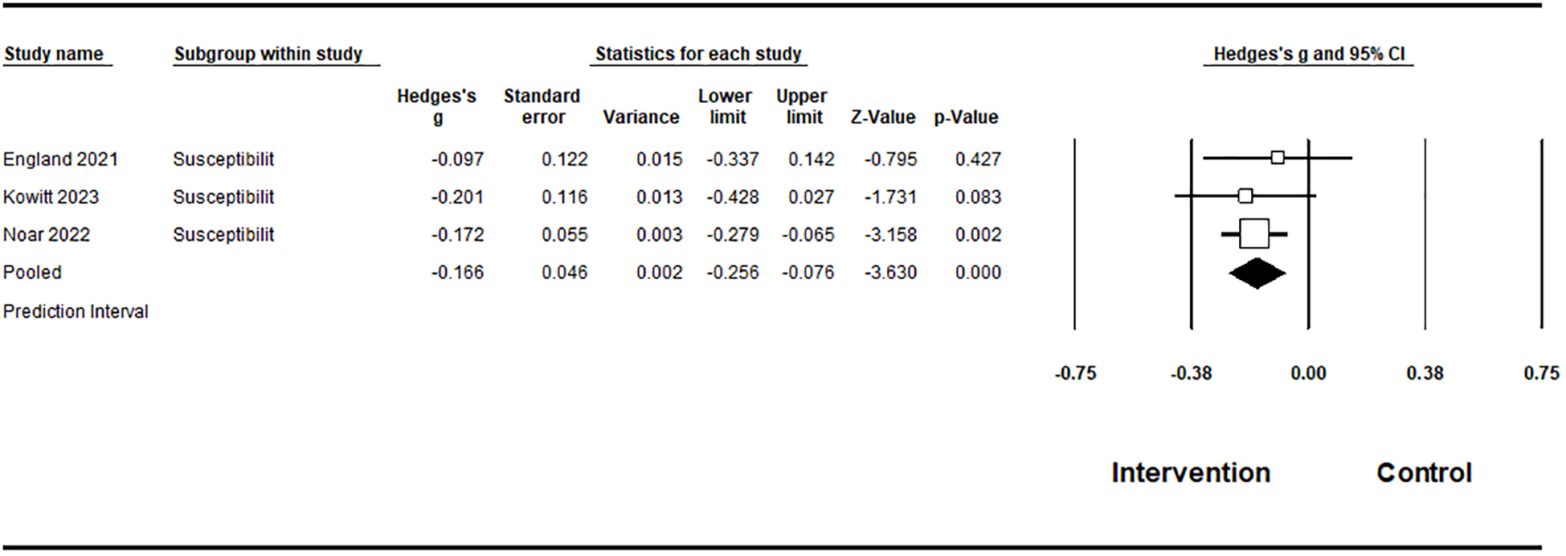

## Discussion

Among youth, the belief that vaping is less harmful than smoking, or even harmless, especially due to flavored products, is widespread. Golan et al. (66) found that many adolescents perceive vaping products to be significantly less harmful than combustible tobacco. Because such misperceptions may shape attitudes and risk beliefs, evaluating campaign-related messages is important for understanding whether they improve vaping-related knowledge and perceptions. Our systematic review indicated that several included studies reported increases in vaping-related knowledge and shifts toward more negative attitudes following exposure to prevention messages. Supporting this, Ma et al. (13) reported that such messages significantly increased vaping-related knowledge. Their findings are broadly consistent with the direction of effects observed in our review for knowledge-related outcomes. Mylocopos et al. (32) similarly reported that social media-based prevention messages can improve knowledge-related outcomes among youth. DiCasmirro et al. (67) also highlighted those interventions included in their scoping review successfully increased knowledge, underlining the importance of social media platforms as key channels, especially given that American adolescents spend an average of eight hours per day on these platforms (68). However, it is important to note that effects were not uniform across studies in our review. Sutfin et al. (51) found no significant improvements in knowledge or attitudes their intervention. The authors attributed this to short intervention duration, limited exposure, and the saturated advertising environment. They recommended that future campaigns prioritize longer duration, greater visibility, and more engaging content to improve effectiveness. Taken together, the pattern of findings suggests that campaign-related messages may shift knowledge and attitudes, which may co-occur with changes in risk perception; however, the temporal and mediating relationships among these outcomes were not directly tested across studies.

In our meta-analysis, exposure to campaign-related prevention messages was associated with a statistically significant, moderate increase in perceived vaping-related risk (g = 0. 254), with effects consistently favoring the intervention. This finding indicates that, under experimental exposure conditions, campaign-related messages are associated with higher perceived harm. Consistent with our findings, Ma et al. (13) reported that exposure to vaping prevention messages significantly increased harm perceptions, reinforcing the role of targeted messaging in shaping risk beliefs. Additional studies report similar directional findings for harm perceptions. Struik et al. (30) identified that behavior change-oriented vaping prevention strategies implemented in North America were particularly effective in enhancing adolescents’ risk perceptions. Likewise, in a study conducted by Trigg et al. (69) adolescents living in South Australia, the campaign materials developed were perceived as likely to enhance youth awareness of vaping harms. Walker et al. (70) also reinforced that adolescents exposed to prevention campaigns demonstrated higher levels of perceived harm and evaluated health and addiction risks more seriously. However, adolescents in today’s digital landscape may also be frequently exposed to pro-vaping advertisements, particularly on social media. Yang et al. (71) found in their meta-analysis that when risk perception is low, particularly in the context of social media, vaping advertisements may increase vaping tendencies among youth. The authors cautioned that frequent exposure to pro-vaping content in such environments may reduce perceived risk and normalize vaping behaviors. While that evidence focuses on promotional content, our review synthesized prevention-oriented message exposure. Overall, the available evidence, including our pooled estimate, suggests prevention-oriented message exposure is associated with higher perceived risk, though effects may vary by context and implementation.

The PME of vaping prevention efforts is a key indicator in understanding their influence on cognitive and behavioral outcomes. Across studies assessing PME and related constructs, campaign-related messages emphasizing health harms and addiction tended to be rated as more effective. These results are consistent with Boynton et al. (72) those who found that traditional themes such as addiction, harmful chemicals, and negative health effects were associated with higher PME, while the use of terms like ‘teen,’ first-person language, and flavor-related content was linked to lower PME in their analysis. Similarly, Ma et al. (13) reported in their meta-analysis that vaping prevention messages significantly increased perceived message effectiveness, influencing cognitive processing, attention, and persuasion. Trigg et al. (69) further emphasized that messages focusing on harmful ingredients and adverse health outcomes are more effective in increasing awareness. They also recommended using individualized, technology-based strategies over youth-targeted slang to reach adolescents more effectively. These findings underscore the relevance of message design for perceived effectiveness, while recognizing that PME and related responses are proximal and do not by themselves demonstrate sustained behavioral change.

Cognitive outcomes (e.g., knowledge and risk perception) are plausible mechanisms targeted by prevention messages. Our synthesis reveals a critical nuance in how these cognitive shifts influence behaviorally proximal indicators: while prevention messages successfully and consistently reduced general susceptibility to vaping, they struggled to significantly alter firm behavioral intentions. This discrepancy can be understood through the lens of the intention-behavior gap, a well-documented phenomenon in organizational and health psychology indicating that cognitive shifts and heightened risk appraisals often fail to translate into definitive behavioral resistance (34, 73). In the context of e-cigarette use, changing a complex behavior requires more than just altering beliefs about harm (34). Susceptibility, often reflecting a passive curiosity among non-users, can be effectively diminished by brief, risk-focused messages. Conversely, behavioral intention represents a firmer commitment that is deeply rooted in subjective norms and peer influence (20, 74). When adolescents are constantly exposed to pro-vaping content on social media (75), the cognitive gains from a short prevention campaign are often overshadowed by the pervasive social acceptability of vaping, rendering their intentions highly resistant to change. Our distinct findings regarding susceptibility and intentions also help contextualize contrasting evidence from the broader literature. For instance, Ma et al. (13) reported small effects on intentions in their meta-analysis, which differs from our non-significant pooled estimate for this specific construct. Their findings underscore the influence of message design on behavioral intentions, suggesting that specific thematic elements may be required to overcome the intention-behavior gap. Conversely, our robust findings on susceptibility align closely with observational longitudinal evaluations, such as the study of Vermont’s “Unhyped” campaign by Glasser et al. (76), which reported lower susceptibility to trying e-cigarettes at a one-year follow-up among adolescents aware of the campaign. While Mylocopos et al. (32) previously concluded that overall syntheses lacked consistent effects on vaping initiation and susceptibility, the present study demonstrates that when susceptibility is isolated from crystallized intentions, prevention messages do exert a significant protective effect. Moving forward, rather than merely assessing whether cognitive shifts organically translate into behavioral changes, future research should prioritize evaluating multi-component interventions. As recent evidence emphasizes, targeting the specific psychosocial predictors of vaping intentions, such as peer norms and social acceptability, is crucial for bridging the intention-behavior gap (23, 77). Therefore, future campaigns and longitudinal studies must focus on identifying which specific message designs and socio-behavioral resistance strategies can effectively convert initial reductions in susceptibility into long-term behavioral resistance.

### Implications for policy, practice, and research

To maximize the public health impact of our findings, we outline specific and actionable implications tailored for policymakers, practitioners, and the scientific community.

For Policymakers: The robust effect of prevention messages on susceptibility to vaping underscores the value of sustained funding for public health campaigns. However, it is critical to note that all included experimental studies in this synthesis were conducted in the United States. Given that youth exposure to pro-vaping content on social media transcends national borders, policymakers must advocate for globally coordinated, social media-based prevention campaigns. Particularly in a European context, where regulatory frameworks like the Tobacco Products Directive (TPD) exist, standardizing strict digital marketing regulations and cross-border public health messaging can serve as vital structural complements to educational campaigns (33, 44).

For Practitioners: Our study findings also have direct practical and clinical applications for local officials, school boards, community-based stakeholders, and point-of-care providers. Relying solely on the didactic communication of health risks is insufficient to bridge the intention-behavior gap (34). Instead, prevention curricula and early-career health professionals who routinely discuss prevention with youth must be equipped to guide adolescents beyond mere risk comprehension. By integrating social resistance strategies into classrooms and clinical encounters, such as discussing peer pressure, deconstructing pro-vaping social media portrayals (75), and actively helping youth develop concrete refusal skills, practitioners can more effectively translate adolescents’ reduced susceptibility into firm, long-lasting intentions to abstain from vaping (45, 78).

For Scientists: For the scientific community, our findings highlight a critical methodological imperative: future evaluations must distinctly separate passive susceptibility from crystallized behavioral intentions to avoid masking specific variance. Furthermore, because current evidence relies exclusively on US-based samples, future longitudinal and experimental studies must be conducted in more diverse global populations. Researchers should prioritize evaluating multi-component interventions that target the specific psychosocial predictors of vaping intentions, such as peer norms and social acceptability, to identify strategies that effectively convert initial cognitive shifts into long-term behavioral resistance (23, 77).

## Conclusion

This systematic review and meta-analysis thoroughly assessed the impact of vaping prevention campaign messages targeting adolescents and young adults on their cognitive and behavioral outcomes. Our systematic review findings indicate that these campaigns had significant positive effects, particularly on cognitive factors such as knowledge level, risk perception, and PME. Notably, the meta-analysis on risk perception revealed a moderate and statistically significant effect, suggesting that prevention campaign messages are associated with increased awareness of vaping-related harms among adolescents. In terms of behaviorally proximal outcomes (susceptibility and intention to vape), although many studies in the systematic review reported positive results, our meta-analytic findings did not support a statistically significant effect. Taken together, these findings indicate that campaign-related message exposure reliably influences cognitive outcomes, whereas evidence for consistent effects on behaviorally proximal outcomes remains limited and heterogeneous. Given that this meta-analysis found consistent effects on cognitive outcomes but small and heterogeneous effects on behaviorally proximal outcomes under predominantly short, controlled exposure conditions, future prevention efforts may benefit from longer-term and repeated message delivery strategies to better support downstream behavioral change. Additionally, greater emphasis should be placed on evaluating cognitive mediators such as perceived message effectiveness and on conducting longitudinal studies that assess the long-term impact of these interventions to achieve more meaningful public health outcomes. In this context, the quality and content of campaign messages hold particular importance; many studies in our review showed that clear messaging, particularly those emphasizing health risks and addiction, was perceived as more effective by adolescents. Therefore, prevention campaigns should be designed not merely to deliver information but as comprehensive interventions that strengthen risk perception and support conditions under which behavioral change may occur.

### Limitation

This systematic review and meta-analysis have limitations. High heterogeneity suggests that differences in study design, sample characteristics, intervention types, and outcomes may affect the reliability and generalizability of effect sizes. Additionally, the limited number of studies available for the separate meta-analyses of behaviorally proximal outcomes precluded formal statistical testing for publication bias, meaning the potential for such bias cannot be definitively ruled out. Participant age ranges varied: most studies focused on adolescents (13–17) and young adults (18–25), but one extended to 29 years, complicating age-specific interpretation; age-stratified analyses were not feasible due to limited data among participants over 25. All included studies were conducted in the United States, limiting the generalizability of findings to other countries with different vaping behaviors, regulatory environments, media landscapes, and cultural norms. Most studies relied on controlled experimental designs, primarily randomized trials, which do not capture real-world factors such as exposure frequency, message fatigue, or environmental saturation. Consequently, findings mainly reflect the effects of campaign-related messages tested under experimental conditions rather than fully implemented, population-level campaigns. Moreover, most studies assessed short-term or single-exposure effects and focused on behaviorally proximal outcomes rather than actual vaping behavior, restricting conclusions about sustained population-level impact. Evidence among young adults was also more limited than among adolescents. Future research should include longer follow-up periods, more diverse populations, and hybrid designs to better evaluate vaping prevention campaigns.

## Data Availability

The raw data supporting the conclusions of this article will be made available by the authors, without undue reservation.
