# Supplementary material for: Effectiveness of vaping prevention campaign messages on cognitive and behaviorally proximal outcomes among adolescents and young adults: a systematic review and meta-analysis
Source: Front Psychiatry. 2026 May 4;17:1796793. doi: 10.3389/fpsyt.2026.1796793 (PMC13180738; doi:10.3389/fpsyt.2026.1796793)
Supplement: Supplementary file 1 [file Table1.docx]

**File S1.** Database-Specific Search Strategies and Number of Records Retrieved

| **Database** | **Search Strategy** | **Records Retrieved** |
| --- | --- | --- |
| **PubMed** | **#1** adolescent[Title/Abstract] OR Teenager[Title/Abstract] OR Teen[Title/Abstract] OR Youth[Title/Abstract] OR Teen[Title/Abstract] OR teenage*[Title/Abstract] OR "Young Adult"[Title/Abstract] "adolescent"[Title/Abstract] OR "Teenager"[Title/Abstract] OR "Teen"[Title/Abstract] OR "Youth"[Title/Abstract] OR "Teen"[Title/Abstract] OR "teenage*"[Title/Abstract] OR "Young Adult"[Title/Abstract]  **#2** vaping[Title/Abstract] OR Ecigarette[Title/Abstract] OR ECig[Title/Abstract] OR Vape[Title/Abstract] OR "E-Cigarette"[Title/Abstract] OR "E-Cig"[Title/Abstract] OR "Electronic Cigarette"[Title/Abstract] "vaping"[Title/Abstract] OR "Ecigarette"[Title/Abstract] OR "ECig"[Title/Abstract] OR "Vape"[Title/Abstract] OR "E-Cigarette"[Title/Abstract] OR "E-Cig"[Title/Abstract] OR "Electronic Cigarette"[Title/Abstract]  **#3** advert*[Title/Abstract] OR "Mobile Social Media"[Title/Abstract] OR "social media"[Title/Abstract] OR "mass media"[Title/Abstract] OR "Broadcast Media"[Title/Abstract] OR "Printed Media"OR "Ads"[Title/Abstract] "advert*"[Title/Abstract] OR "Mobile Social Media"[Title/Abstract] OR "social media"[Title/Abstract] OR "mass media"[Title/Abstract] OR "Broadcast Media"[Title/Abstract] OR "Printed Media"[All Fields] OR "Ads"[Title/Abstract]  **#4** ((adolescent[Title/Abstract] OR Teenager[Title/Abstract] OR Teen[Title/Abstract] OR Youth[Title/Abstract] OR Teen[Title/Abstract] OR teenage*[Title/Abstract] OR "Young Adult"[Title/Abstract]) AND (vaping[Title/Abstract] OR Ecigarette[Title/Abstract] OR ECig[Title/Abstract] OR Vape[Title/Abstract] OR "E-Cigarette"[Title/Abstract] OR "E-Cig"[Title/Abstract] OR "Electronic Cigarette"[Title/Abstract])) AND (advert*[Title/Abstract] OR "Mobile Social Media"[Title/Abstract] OR "social media"[Title/Abstract] OR "mass media"[Title/Abstract] OR "Broadcast Media"[Title/Abstract] OR "Printed Media"OR "Ads"[Title/Abstract]) ("adolescent"[Title/Abstract] OR "Teenager"[Title/Abstract] OR "Teen"[Title/Abstract] OR "Youth"[Title/Abstract] OR "Teen"[Title/Abstract] OR "teenage*"[Title/Abstract] OR "Young Adult"[Title/Abstract]) AND ("vaping"[Title/Abstract] OR "Ecigarette"[Title/Abstract] OR "ECig"[Title/Abstract] OR "Vape"[Title/Abstract] OR "E-Cigarette"[Title/Abstract] OR "E-Cig"[Title/Abstract] OR "Electronic Cigarette"[Title/Abstract]) AND ("advert*"[Title/Abstract] OR "Mobile Social Media"[Title/Abstract] OR "social media"[Title/Abstract] OR "mass media"[Title/Abstract] OR "Broadcast Media"[Title/Abstract] OR "Printed Media"[All Fields] OR "Ads"[Title/Abstract]) | **742** |
| **PubMed** | #Supplementary Search: ("Electronic Nicotine Delivery Systems"[Title/Abstract] OR ENDS[Title/Abstract]) AND (adolescent[Title/Abstract] OR teenager[Title/Abstract] OR teen[Title/Abstract] OR youth[Title/Abstract] OR "young adult"[Title/Abstract]) AND (advert*[Title/Abstract] OR "social media"[Title/Abstract] OR "mass media"[Title/Abstract] OR campaign[Title/Abstract] OR message*[Title/Abstract]) | 2 |
| **Cochrane Library** | **#1** adolescent OR Teenager OR Teen OR Youth OR Teen OR teenage* OR "Young Adult"  **#2** vaping OR Ecigarette OR ECig OR Vape OR "E-Cigarette" OR "E-Cig" OR "Electronic Cigarette"  **#3** advert* OR "Mobile Social Media" OR "social media" OR "mass media" OR "Broadcast Media" OR "Printed Media" OR "Ads"  #1 AND #2 AND #3 | 115 |
| **Scopus** | TITLE-ABS ( adolescent OR teenager OR teen OR youth OR teen OR teenage* OR "Young Adult" ) AND TITLE-ABS ( vaping OR ecigarette OR ecig OR vape OR "E-Cigarette" OR "E-Cig" OR "Electronic Cigarette" ) AND TITLE-ABS ( advert* OR "Mobile Social Media" OR "social media" OR "mass media" OR "Broadcast Media" OR "Printed Media" OR "Ads" ) | 1045 |
| **Web of Science** | **#1** (TI=(adolescent OR Teenager OR Teen OR Youth OR Teen OR teenage* OR "Young Adult")) OR AB=(adolescent OR Teenager OR Teen OR Youth OR Teen OR teenage* OR "Young Adult")  **#2** (TI=(vaping OR Ecigarette OR ECig OR Vape OR "E-Cigarette" OR "E-Cig" OR "Electronic Cigarette")) OR AB=(vaping OR Ecigarette OR ECig OR Vape OR "E-Cigarette" OR "E-Cig" OR "Electronic Cigarette")  **#3** (TI=(advert* OR "Mobile Social Media" OR "social media" OR "mass media" OR "Broadcast Media" OR "Printed Media" OR "Ads")) OR AB=(advert* OR "Mobile Social Media" OR "social media" OR "mass media" OR "Broadcast Media" OR "Printed Media" OR "Ads")  #1 AND #2 AND #3 | 514 |
| **CINAHL** | #1 TI ( adolescent OR Teenager OR Teen OR Youth OR Teen OR teenage* OR "Young Adult" ) OR AB ( adolescent OR Teenager OR Teen OR Youth OR Teen OR teenage* OR "Young Adult" ) Expanders - Also search within the full text of the articles; Apply equivalent subjects  **#2** TI ( vaping OR Ecigarette OR ECig OR Vape OR "E-Cigarette" OR "E-Cig" OR "Electronic Cigarette" ) OR AB ( vaping OR Ecigarette OR ECig OR Vape OR "E-Cigarette" OR "E-Cig" OR "Electronic Cigarette" ) Expanders - Also search within the full text of the articles; Apply equivalent subjects  **#3** TI ( advert* OR "Mobile Social Media" OR "social media" OR "mass media" OR "Broadcast Media" OR "Printed Media" OR "Ads" ) OR AB ( advert* OR "Mobile Social Media" OR "social media" OR "mass media" OR "Broadcast Media" OR "Printed Media" OR "Ads" ) Expanders - Also search within the full text of the articles; Apply equivalent subjects  #1 AND #2 AND #3 | 189 |
| **Total** |  | **2607** |
| **After deduplication** |  | **810** |
